# Metastatic BRAF K601E-mutated melanoma reaches complete response to MEK inhibitor trametinib administered for over 36 months

**DOI:** 10.1186/s40164-017-0067-4

**Published:** 2017-03-21

**Authors:** Riccardo Marconcini, Luca Galli, Andrea Antonuzzo, Simona Bursi, Claudia Roncella, Gabriella Fontanini, Elisa Sensi, Alfredo Falcone

**Affiliations:** 1 Department of Oncology, Azienda Ospedaliero-Universitaria Pisana and University of Pisa, Istituto Toscano Tumori, Santa Chiara Hospital, Via Roma 67, 56100 Pisa, Italy; 2 Department of Oncology, Ospedale Civile-Istituto Toscano Tumori, Livorno, Italy; 3 Diagnostic and Interventional Radiology, University of Pisa, Pisa, Italy; 4 Units of Pathological Anatomy, Department of Surgical, Medical, Molecular Pathology and Critical Area, University of Pisa, Pisa, Italy

**Keywords:** BRAF K601E mutation, Complete response, Melanoma, Trametinib

## Abstract

**Background:**

The BRAF K601E mutation occurs in 5% of patients with melanoma, and is the third most common type of BRAF mutation. However, treatment with BRAF and mitogen-activated extracellular signal-regulated kinase (MEK) inhibitors is only approved in patients with BRAF V600-positive melanoma, and patients with K601E-mutated melanoma do not have access to such drugs.

**Case presentation:**

A female patient was diagnosed with high tumor burden metastatic melanoma harboring the BRAF K601E mutation. After chemotherapy failure, she underwent compassionate treatment with trametinib. Trametinib showed good activity and efficacy, with 48% shrinkage of a metastatic lymphadenopathy after 4 months’ treatment. However, the patient reported treatment-related skin toxicity that required dosage reduction and a personalized intermittent trametinib dosing schedule. After over 36 months from the first trametinib administration, and resection of a metastatic lymphadenopathy, the patient experienced complete response.

**Conclusions:**

This case report shows that trametinib could be a valid therapeutic option in patients with metastatic melanoma harboring the rare BRAF K601E mutation.

## Background

Melanoma is the fourth most common malignancy in men and women. Efficacious targeted therapies such as BRAF and mitogen-activated extracellular signal-regulated kinase (MEK) inhibitors are available for patients with melanoma harboring BRAF V600 mutations. Reported BRAF mutation rates are between 40 and 60, and >90% of these are due to V600 mutations, in particular V600E and V600K [1, 2]. A small proportion of BRAF-mutated melanomas harbor mutations at codon K601 in exon 15 of the BRAF gene. This mutation results in an amino acid substitution from a lysine (K) to a glutamic acid (E) at position 601 in BRAF, and consequently elevated kinase activity [2]. BRAF inhibitors (e.g. vemurafenib, dabrafenib) and MEK inhibitors (e.g. trametinib, cobimetinib) have been shown to be effective in providing a rapid tumor response, prolongation of progression-free survival, and improving overall survival in BRAF V600-mutated melanoma [3, 4]. Less common mutations may have clinical relevance as there is preliminary evidence of sensitivity to targeted therapies [5], but data published in this regard are limited compared with what is known about common BRAF V600 mutations. We describe a case report of a patient with BRAF K601E-mutated melanoma who achieved complete response to the MEK inhibitor trametinib, that underlines a possible therapeutic option for these patients.

## Case presentation

A 60-year old female patient, with no relevant comorbidities or family history of melanoma, underwent an excision of an infrascapular skin lesion of the back in July 2012. Histologic examination revealed a superficial spread of melanoma (Breslow 3.46 mm, Clark IV, number of mitosis: 13 mitosis/mm^3^, ulceration absent, negative margins).

In August 2012, an enlargement of surgical margins and sentinel lymph node research were performed; one right and one left axillary lymph node were removed, and were negative for melanoma metastases. Stadiative radiological examination was also negative for metastatic disease, and a follow-up program was started, in accordance with the patient’s decision not to undergo adjuvant interferon treatment. Molecular analysis of the primary tumor was positive for the K601E BRAF mutation (nucleotide substitution c. 1801 A > G).

**Fig. 1:**
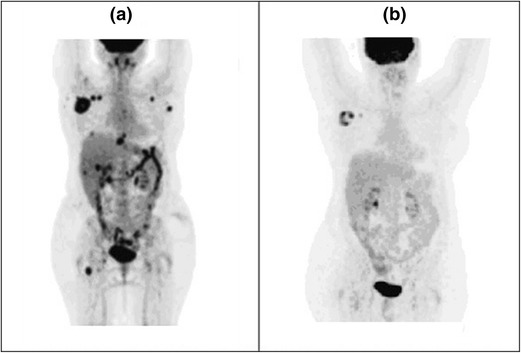
Fluorodeoxyglucose positron emission tomography (FDG-PET) performed in different times are shown: **a** FDG-PET performed in March 2013 (before trametinib treatment); **b** FDG-PET performed in May 2015 after 20 months of trametinib treatment

During follow-up in March 2013, ultrasonographic examination evidenced right axillary lymphadenopathy. A computed tomography (CT) scan with contrast showed right axillary lymph node metastases and liver lesions, and a full-body fluorodexoyglucose positron emission tomography (FDG-PET) scan confirmed the presence of metastases in the right axillary lymph node and hepatic segment V; metastases in left axillary, rear left scapular, right retroperitoneal, skeletal D8–D9, and vertebral L1 regions were also observed (Fig. 1a).

Due to the absence of the BRAF V600 mutation, no targeted therapies were available at that time, and there were no clinical trials enrolling patients with BRAF K601E-mutated melanoma in our centers. Considering the extent and rapid spread of the disease, the patient was a candidate for chemotherapy. From May 2013 to July 2013, the patient received first-line chemotherapy according to the following schedule: cisplatin 75 mg/m^2^ i.v. day 1 plus dacarbazine 800 mg/m^2^ i.v. day 1, administered every 21 days.

**Fig. 2:**
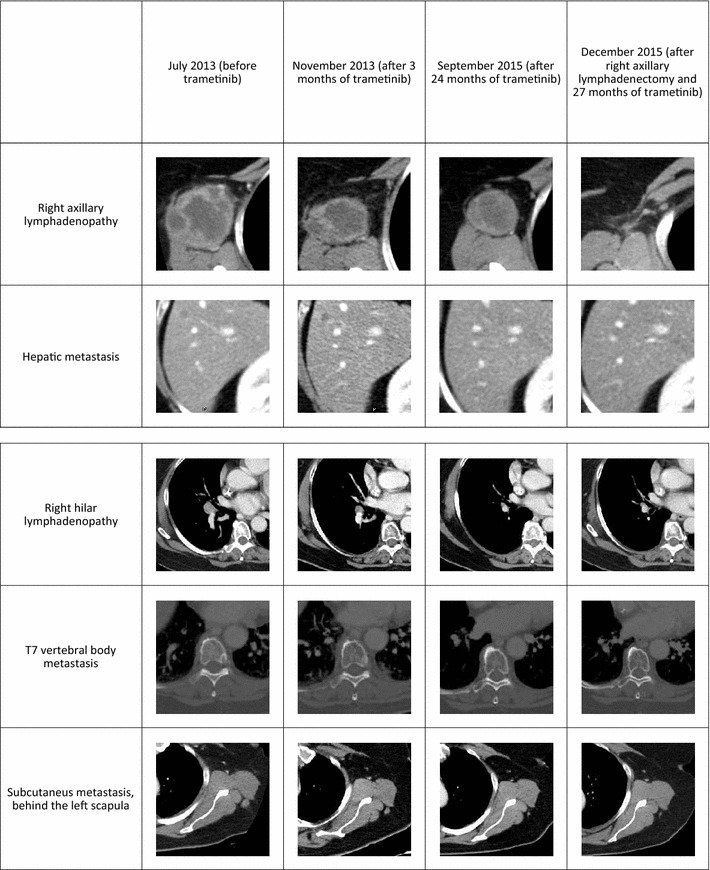
Computed tomography (CT) scan performed during trametinib treatment: images showing *right* axillary lymphadenopathy, liver metastasis, *right* hilar lymphadenopathy, T7 vertebral body metastasis and subcutaneous node are shown

After the fourth cycle, in July 2013, a total-body CT scan with contrast showed disease progression. The previous lesions were confirmed, and the scan additionally revealed an increased number of lymphadenopathies in the right axillary region (maximum diameter 65 mm), a millimetric lesion in the right hepatic lobe, two new metastatic lesions in the precharinal and right lung ilus (maximum diameter 16 mm), and new bone metastases in the left rib IX and right acetabulum (Fig. 2a).

In consideration of the patient’s disease stage, previous treatment, and the BRAF K601E mutational status for which treatment with BRAF inhibitors was not approved, compassionate treatment with continuous oral trametinib 2 mg once daily was required. However, while waiting for the delivery of trametinib, the patient’s general condition deteriorated and second-line chemotherapy was started with paclitaxel 80 mg/m^2^ i.v. weekly; this was administered for 4 weeks until 21 August 2013. In September 2013, the patient started continuous treatment with trametinib.

Due to persistent low back pain, radiotherapy in the D8 vertebra was administered from 14 to 24 September 2013 (total dose 30 Gy; 10 fractions of 3 Gy), with resolution of pain.

In November 2013, the patient reported trametinib-related grade 3 erythema with pruritus that extended to the upper part of the body and required discontinuation of trametinib and symptomatic therapy with local corticosteroid and antihistamines. Ten days after treatment discontinuation, following resolution of the skin toxicity, continuous oral trametinib was restarted at a lower dosage (1.5 mg/day).

In December 2013, a CT scan showed partial response, with reduction of the right axillary lesion (to 34 mm diameter), precarinal lesion (to 9 mm diameter), and subcutaneous retroscapular lesion (to 9 mm diameter) (Fig. 2b).

From 22 January 2014 to 5 March 2014, trametinib administration was interrupted due to a new episode of grade 3 skin toxicity that persisted for more than 2 weeks and slowly resolved with corticosteroids and antihistamines. Continuous trametinib therapy was then restarted at a reduced dosage of 1 mg/day. In March 2014, a CT scan confirmed response to treatment, with no evidence of metastases in the liver or bones and disappearance of the precarinal and subcutaneous retroscapular lesions; the only remaining lesion was the right axillary lymphadenopathy. In a CT scan performed in June 2014, the response was maintained.

Due to grade 1 asthenia, articular pain in the ankles, knees, and wrists, and the earlier grade 3 skin toxicities, the patient received symptomatic treatment and the schedule of trametinib therapy was modified to an on/off regimen: 2 weeks of trametinib 1 mg/day, followed by 1 week of no trametinib administration. With this new schedule, the patient experienced no adverse effects and was able to continue treatment with trametinib.

Subsequent CT scans (performed every 3–4 months) showed maintenance of complete response in all known lesions, with the exception of the increasing lymphadenopathy in the right axilla (Fig. 2c). Also, in May 2015, an FDG-PET scan showed complete response in every metastatic site, with the exception of the right axillary lymphadenopathy. In October 2015, the patient underwent excision of the right axillary lymphadenopathy, and histologic examination revealed a large melanoma metastasis. BRAF and NRAS mutational analysis of the lymphadenopathy metastasis revealed BRAF K601E mutation and NRAS wild-type status.

In December 2015, a new CT scan confirmed complete response, with no evaluable lesions (Fig. 2d). The patient continued trametinib treatment at the same dose. These findings were confirmed in the last CT scan performed in September 2016. The patient continues to receive trametinib with a personalized on/off schedule.

## Discussion

This case report demonstrates the activity and efficacy of the MEK inhibitor trametinib in BRAF K601E-mutated melanoma. The BRAF K601E mutation occurs in 5% of patients with melanoma, and it is the third most common BRAF mutation [1, 2]. Treatment with a BRAF inhibitor is only approved in patients with BRAF V600-positive melanoma, and is not active in patients with other mutations. However, the BRAF K601E mutation is able to hyperactivate the BRAF protein, causing increased activity in the mitogen-activated protein (MAP) kinase pathway. As the MEK protein is located in the MAP kinase pathway beyond the BRAF protein, it is also hyperactivated by BRAF mutations [6]. Therefore, there is a rationale for using a MEK inhibitor in a patient with BRAF K601E-mutated melanoma.

Trametinib is a MEK inhibitor that acts by blocking MEK molecules within the MAP kinase signaling pathway, which mediates cell proliferation and survival, and is often deregulated in cancer cells [7–12]. Boyer and colleagues have demonstrated that in patients with BRAF K601E-mutated melanoma, treatment with trametinib can reduce the activity of extracellular signal-regulated kinase (another MAP kinase protein located beyond MEK in the MAP kinase pathway), corresponding to further activity of trametinib against disease [5].

To the best of our knowledge, this the first report of trametinib treatment in a patient with BRAF K601E-mutated melanoma who achieved complete response after more than 36 months. Our case report confirms that trametinib has a high levels of activity against BRAF K601E-mutated melanoma, with our patient experiencing dramatic reduction of tumor burden and an increased performance status. The patient’s initial tumor burden was large, with many organs involved and metastasis and lesions that reached a maximum of 6.5 cm in diameter. In addition, the patient had previously received chemotherapy. Therefore, this case report showed trametinib activity regardless of tumor size or previous treatments, suggesting that in the presence of the BRAF K601E mutation, trametinib can be used independently of these factors.

Due to skin toxicities, the patient’s trametinib administration required treatment interruption. It is thought that BRAF inhibitor therapy should be administered continuously in order to prevent the onset of drug resistance. However, data from a preclinical study in mice [13] and a case series of six patients [14] suggest that intermittent BRAF inhibitor dosing may delay drug resistance, while continuous BRAF inhibitor dosing does not. Data regarding the efficacy of intermittent MEK inhibitor dosing are lacking. This case report is an example of the maintained activity of trametinib when used with an intermittent dosing schedule.

The toxicities experienced by the patient in this report were mainly cutaneous, reached grade 3 severity, and required dosage reductions until a personalized on/off intermittent dosing schedule was started. With these treatment modifications and adequate use of symptomatic therapy, trametinib treatment was subsequently not interrupted, and was finally well tolerated by the patient.

After 2 years of treatment, the patient underwent surgery to excise the only metastatic site that demonstrated progression during trametinib treatment. The surgery led to complete response and systemic trametinib treatment was continued. This case underlines how a multidisciplinary approach, including targeted therapy and surgery, is necessary in the management of a patient with melanoma.

## Conclusion

This case report demonstrates that trametinib had significant activity and efficacy in treating a patient with BRAF K601E-mutated metastatic melanoma, regardless of tumor burden and previous treatments, maintaining its activity for over 36 months, despite modification of administration to an intermittent dosing schedule.

## Abbreviations

CT: computed tomography
MAP: mitogen-activated protein
MEK: mitogen-activated extracellular signal-regulated kinase
